# Numerical Study and Optimization of a Novel Piezoelectric Transducer for a Round-Window Stimulating Type Middle-Ear Implant

**DOI:** 10.3390/mi10010040

**Published:** 2019-01-09

**Authors:** Houguang Liu, Hehe Wang, Zhushi Rao, Jianhua Yang, Shanguo Yang

**Affiliations:** 1 School of Mechatronic Engineering, China University of Mining and Technology, Xuzhou 221116, China; liuhg@cumt.edu.cn (H.L.); jianhuayang@cumt.edu.cn (J.Y.); ysgcumt@cumt.edu.cn (S.Y.); 2 State Key Laboratory of Mechanical System and Vibrations, Shanghai Jiao Tong University, Shanghai 200240, China; zsrao@sjtu.edu.cn

**Keywords:** piezoelectric transducer, middle ear implant, round window stimulation, hearing loss, flextensional amplifier, finite element analysis

## Abstract

Round window (RW) stimulation is a new application of middle ear implants for treating hearing loss, especially for those with middle ear disease. However, most reports on it are based on the use of the floating mass transducer (FMT), which was not originally designed for round window stimulation. The mismatch of the FMT’s diameter and the round window membrane’s diameter and the uncontrollable preload of the transducer, leads to a high variability in its clinical outcomes. Accordingly, a new piezoelectric transducer for the round-window-stimulating-type middle ear implant is proposed in this paper. The transducer consists of a piezoelectric stack, a flextensional amplifier, a coupling rod, a salver, a plate, a titanium housing and a supporting spring. Based on a constructed coupling finite element model of the human ear and the transducer, the influences of the transducer design parameters on its performance were analyzed. The optimal structure of the supporting spring, which determines the transducer’s resonance frequency, was ascertained. The results demonstrate that our designed transducer generates better output than the FMT, especially at low frequency. Besides this, the power consumption of the transducer was significantly decreased compared with a recently reported RW-stimulating piezoelectric transducer.

## 1. Introduction

Hearing loss is one of the most common physical disabilities in our society, affecting more than 538 million people worldwide [1]. Despite impressive advances in microsurgical techniques, audiology and biotechnology, the relief we can offer to patients with hearing loss is still inadequate, especially for patients with sensorineural hearing loss [2]. Hearing aids can accomplish partial rehabilitation of sensorineural hearing loss; however, only a minority of patients choose to use them due to their inherent disadvantages, such as limited amplification, acoustic feedback, poor fidelity and the stigma of aging [3]. To overcome these shortcomings of conventional hearing aids, many institutions began to develop middle ear implants (MEIs) [4,5,6,7,8] which treat hearing loss directly by their implanted transducer’s mechanical stimulation of the patient’s ossicular chain. However, attaching the transducer to the ossicular chain is difficult in patients with middle ear disease, such as middle ear malformation, chronic otitis media and otosclerosis. To solve this issue, an alternative approach of coupling the transducer to the ear by stimulation of the round window (RW) membrane, called RW stimulation, has been widely investigated [9,10,11].

Although clinical data have confirmed the feasibility of RW stimulation, its outcomes have shown high variability. To determine the cause of these large variations, a number of studies have been conducted. Since the MED-EL Vibrant Soundbridge’s floating mass transducer (FMT)—the transducer that has been widely used clinically for RW stimulation—has a diameter similar to the diameter of the RW, Nakajima et al. concluded that the bony overhang surrounding the RW could hinder the coupling of the FMT’s motion to the RW [12]. Although introducing a compliant coupling layer between the transducer and the RW membrane would improve the vibration transmission to the RW [13], the coupling condition is difficult to control in surgery. Schraven et al. even stated that the high variability in the postoperative outcome of RW stimulation is primarily owing to the success of this coupling [14]. Besides this, Maier et al. performed an experimental study on human temporal bone which indicated a clear dependency of the sound transmission efficiency on the static force applied to the RW membrane [15]. Furthermore, based on an experimental investigation, Muller et al. suggested that the optimal preload of the transducer should less than 20 mN [16]. However, as most transducers used in the clinic are not originally designed for RW stimulation, the preload of the transducer on the round window membrane (RWM) cannot be precisely monitored.

In addition to high output variability, reports also show that using the FMT for RW stimulation has a considerably low output at lower frequencies (< 1 kHz) [17,18]. To address this issue, Shin et al. designed a novel piezoelectric transducer specifically for RW stimulation [19]. In comparison with the widely used electromagnetic transducer, that is, the FMT, their experimental results demonstrated that their transducer has excellent low-frequency output. Moreover, this piezoelectric transducer displays the advantages of ease of fabrication, wider bandwidth and compatibility with an external magnetic environment. However, the preload of the transducer applied to the RW membrane is still difficult to precisely control.

Accordingly, in this paper, we propose a new piezoelectric transducer incorporating a preload visual indicator for RW stimulation. Besides this, to further decrease the power consumption of the piezoelectric transducer, we introduced a flextensional amplifier for the piezoelectric stack in the transducer. Then, based on a coupling finite element model of a human ear and the transducer, the main design parameters of the transducer were optimized to produce sufficient driving force for RW stimulation. The results indicate that the new piezoelectric transducer not only has excellent output over the audio frequency range but also significantly decreases the power consumption.

## 2. Materials and Methods

### 2.1. Concept and Structure of the Piezoelectric Transducer

**Figure 1:**
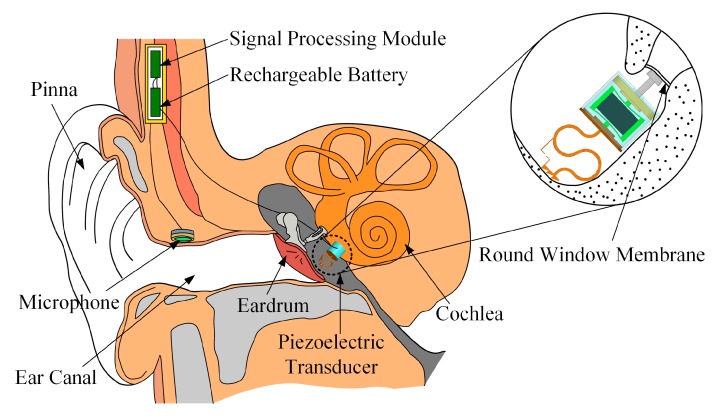
Concept illustration of the round window (RW)-stimulating-type middle ear implant.

Figure 1 shows a schematic view of the proposed round-window-stimulating-type middle ear implant (RW-MEI) in the human ear. This RW-MEI mainly consists of three parts: a piezoelectric transducer, a microphone and an assembly of a signal processing module and a rechargeable battery. The microphone, which is implanted near the entrance of the ear canal to take advantage of the natural sound filtering from the pinna, is responsible for picking up sound from the outside and generating a corresponding electrical signal. Then, the signal is transmitted to the signal processing module, which processes it according to the patient’s individual hearing loss conditions and produces a driving electrical signal for the implanted piezoelectric transducer. Finally, the piezoelectric transducer, which is placed in the middle ear cavity with its tip attached to the round window membrane, converts the electrical signal into a mechanical vibration that is delivered directly to the cochlea. In the cochlea, the vibration is detected by hair cells and perceived as sound.

**Figure 2:**
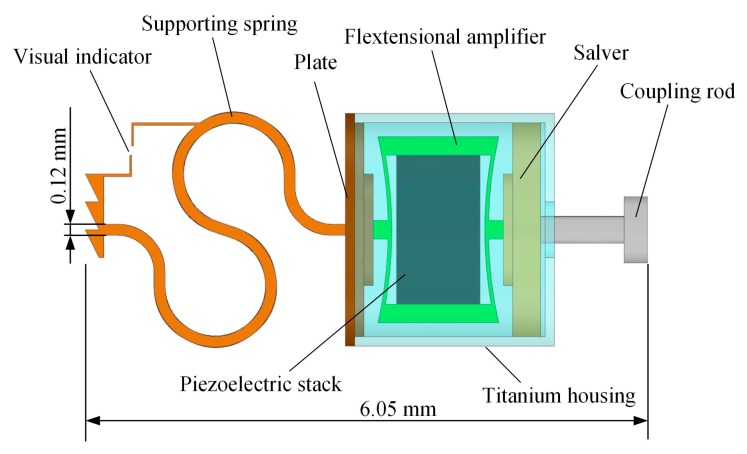
The inner structure of the proposed piezoelectric transducer.

The inner structure of the round-window-stimulating-type piezoelectric transducer is shown in Figure 2. This transducer is composed of a coupling rod, a salver, a piezoelectric component, a titanium housing, a plate and a supporting spring. The piezoelectric component consists of two parts: a piezoelectric stack and a flextensional amplifier. The flextensional amplifier was introduced to amplify the displacement output of the piezoelectric stack, so as to decrease the required driving voltage and reduce the power consumption. To enable the surgeon to detect the preload applied on the RW membrane, a force visual indicator was set on the supporting spring. During the surgical procedure, the transducer is implanted into a bone bed drilled behind the mastoid. The back end of the supporting spring is contacted with the bony wall opposite the RW membrane. The other end of the supporting spring fixes to a plate, which connects with the base side of the piezoelectric components. Biocompatibility of the piezoelectric component is secured by coating its entire surface with biocompatible materials and putting it into a titanium housing. Besides this, according to an experimental study by Arnold et al. [20], the vibration transmission to the cochlea is best when the transducer output is perpendicular to the round window membrane. To ensure the direction of the transducer output, we fixed the transducer’s output coupling rod on a salver, which is settled in the titanium housing and connected the salver with the piezoelectric component. The geometry of the salver’s outer wall is consistent with that of the titanium housing’s inner wall, thereby forming a sliding pair and guiding the output direction of the piezoelectric component along the longitudinal direction of the titanium housing. The tip of the coupling rod attaches to the round window membrane and transmits the transducer’s vibration to the cochlea.

### 2.2. Design of the Piezoelectric Transducer

#### 2.2.1. Design of the Piezoelectric Component

**Figure 3:**
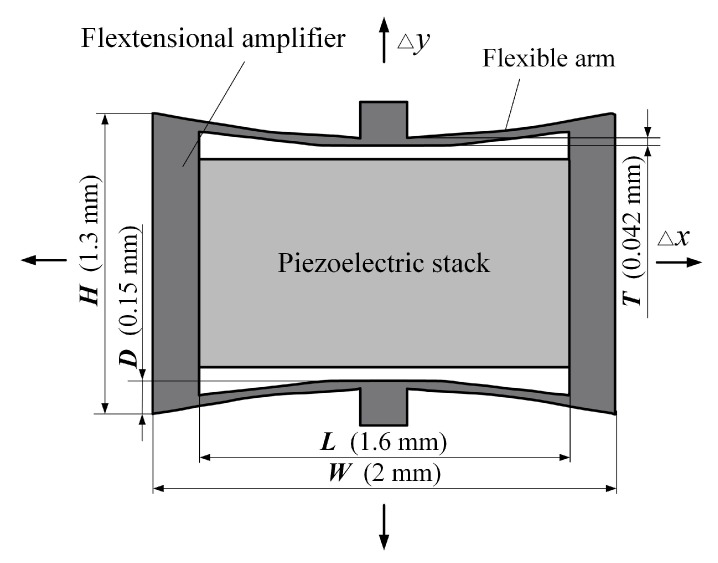
The structure of the piezoelectric component.

The piezoelectric component of this transducer consists of a piezoelectric stack and a flextensional amplifier, as shown in Figure 3. Considering the small space in the human middle ear cavity, where the transducer is implanted, the size of the transducer should be restricted. For comparison, the piezoelectric stack used in this study is the same as that used by Shin et al. [19]. Specifically, this piezoelectric stack is made of lead zirconate titanate ceramics (PZT-8) with dimensions of 0.9 mm × 0.9 mm × 1.6 mm, which is small enough for this application. This piezoelectric stack has a layer number of 222 to produce 300 nm displacement under a voltage of 6 V. The flextensional amplifier was incorporated to further decrease the transducer’s power consumption. As shown in Figure 3, once the piezoelectric stack generates a Δ*x* deformation driven by a voltage, the flextensional amplifier would generate an amplified output displacement: Δ*y*. Their relationship can be expressed as Equation (1), where *G* is the displacement amplification ratio of the flextensional amplifier. The value of *G* can be estimated based on Equation (2):

(1) Δy=GΔx

(2) G=L/2D

where *L* is the length of the amplifier’s inner cavity and *D* is the amplifier’s unilateral inner concave depth. Considering the spatial limitation in the middle ear cavity, the height (*H*), the width (*W*) and the thickness of the amplifier were set to 1.3 mm, 2.0 mm and 0.9 mm, respectively. *L* and *D* were set to 1.6 mm and 0.15 mm. The thickness of the flexible arm was set to 42 μm. According to Equation (2), the obtained amplification ratio is 5.3.

#### 2.2.2. Design of the Supporting Spring

The design of the supporting spring significantly influences the transducer’s resonant frequency. Considering that the middle ear implant is designed for compensating hearing loss, its frequency characteristics should be similar to those of the human middle ear. Based on a human temporal bone experiment, Homma et al. reported that the human middle ear resonant frequency is around 0.8–1.2 kHz [21]; therefore, the resonant frequency of our designed transducer should lie within this frequency range.

**Figure 4:**
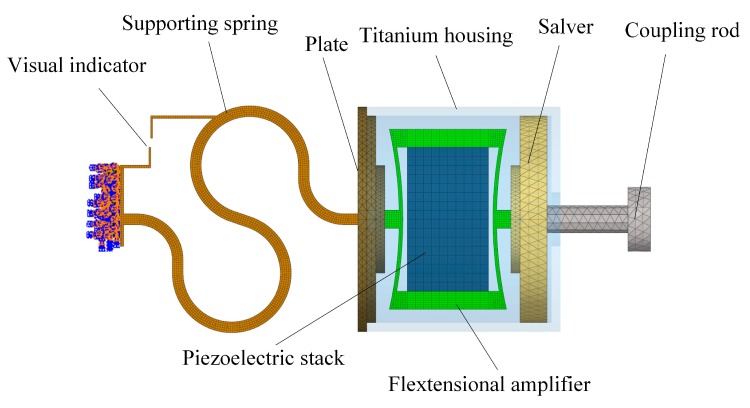
Finite element model of the proposed piezoelectric transducer.

To facilitate the design of this supporting spring, we constructed a finite element model of the piezoelectric transducer, as shown in Figure 4. This finite element model consists of six components: the coupling rod, the salver, the flextensional amplifier, the plate, the titanium housing and the supporting spring. The piezoelectric stack, which is made of PZT-8, was meshed by 1600 hexahedral elements. The density of PZT-8 is 7600 kg/m^3^. The other material properties of it are listed in Table 1. The piezoelectric finite element model equations can be written in terms of the nodal displacement ***U*** and nodal electrical potential ***Φ*** for each node. Under the action of the nodal electric charge ***Q*** and the external mechanical forces ***F***, the equilibrium equations are expressed in matrix form as [22]

(3) $$\left[\begin{array}{cc} M_{uu} & 0\\ 0 & 0 \end{array}\right]\left[\begin{array}{c} \ddot{U}\\ \ddot{\Phi } \end{array}\right]+\left[\begin{array}{cc} C_{uu} & 0\\ 0 & 0 \end{array}\right]\left[\begin{array}{c} \dot{U}\\ \dot{\Phi } \end{array}\right]+\left[\begin{array}{cc} K_{uu} & K_{u\varphi }\\ K_{u\varphi }{}^{T} & K_{\varphi \varphi } \end{array}\right]\left[\begin{array}{c} U\\ \Phi \end{array}\right]=\left[\begin{array}{c} F\\ Q \end{array}\right]$$

with

$$\begin{array}{l} K_{uu}=\underset{\Omega e}{∭}B_{u}{}^{T}cB_{u}dV,\;K_{u\varphi }=\underset{\Omega e}{∭}B_{u}{}^{T}eB_{\varphi }dV,\;K_{\varphi \varphi }=\underset{\Omega e}{∭}B_{\varphi }{}^{T}\varepsilon B_{\varphi }dV\\ M_{uu}=\rho \underset{\Omega e}{∭}N_{u}{}^{T}N_{u}dV,\;C_{uu}=\beta K_{uu} \end{array}$$

denoting the mechanical stiffness matrix, piezoelectric coupling matrix, dielectric stiffness matrix, mass matrix and mechanical damping matrix, respectively.

**Figure 5:**
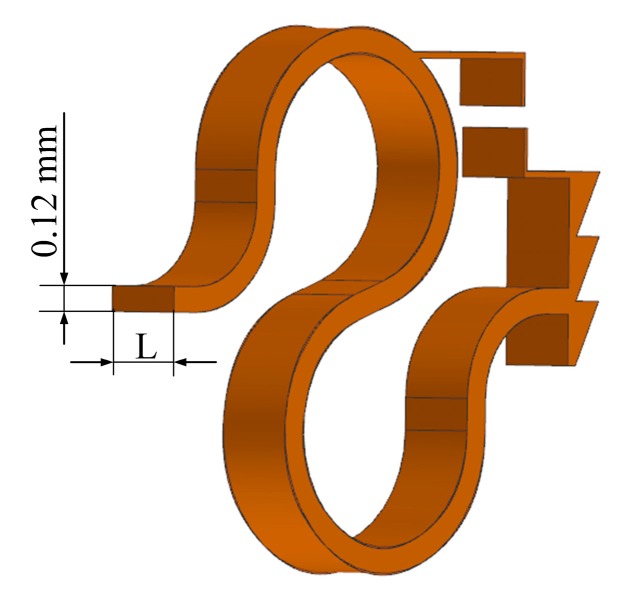
The structure of the supporting spring.

**Figure 6:**
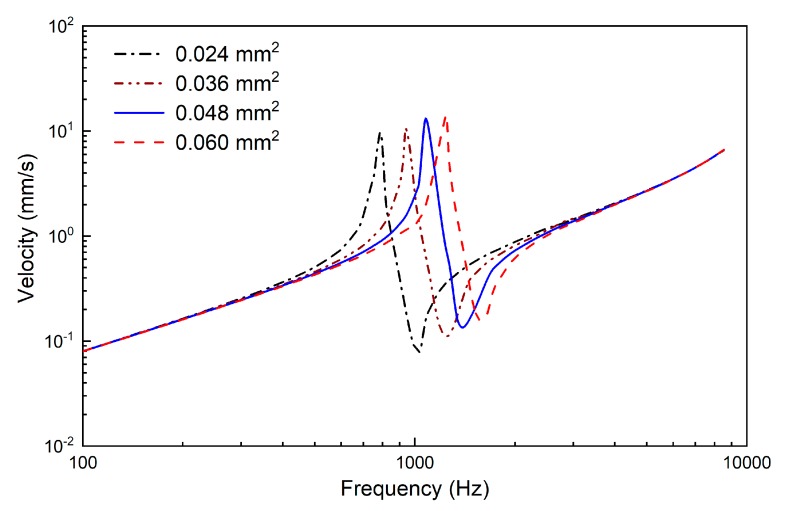
The effect of the supporting spring’s cross-sectional area on the output of the transducer.

All the other parts of the transducer are made of titanium. The corresponding density is 4430 kg/m^3^, the Young’s modulus is 116 GPa and the Poisson’s ratio is 0.32. The flextensional amplifier was meshed by 7680 hexahedral elements. The salver, titanium housing, plate and supporting spring were meshed by 15224, 13076, 13228 and 21144 tetrahedral elements, respectively. The connections between the coupling rod and salver, the salver and the flextensional amplifier, the flextensional amplifier and plate and the plate and the supporting spring were modeled by coupling the corresponding finite element nodes of them. The end of the supporting spring was fixed by defining a fixed constraint on the corresponding nodes. The influence of the supporting spring’s cross-sectional area on the transducer’s frequency characteristics was analyzed by changing the width of its cross section (*L* in Figure 5). The model-predicted transducer output velocity is shown in Figure 6, in which the transducer was driven by a voltage of 0.5 V. It shows that when the cross-sectional area of the supporting spring changes from 0.024 mm^2^ to 0.036 mm^2^, 0.048 mm^2^, or 0.060 mm^2^, the transducer’s resonant frequency changes from 788 Hz to 938 Hz, 1082 Hz, or 1243 Hz, respectively. To make sure that the transducer has a resonant frequency close to 1100 Hz, we selected 0.048 mm^2^ as the cross-sectional area of the supporting spring. Thus, the final width of the supporting spring was 0.4 mm. Based on this geometric size, the static analysis showed that the visual indicator deformed 20 μm under 20 mN; therefore, the width of the two bars on the visual indicator was set to 20 μm to make them coincide when the transducer preload increases to 20 mN.

#### 2.2.3. Design of the Coupling Rod Tip

**Figure 7:**
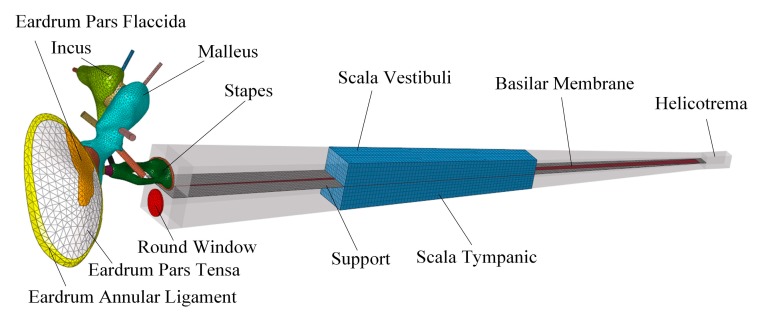
The constructed human ear finite element model.

**Figure 8:**
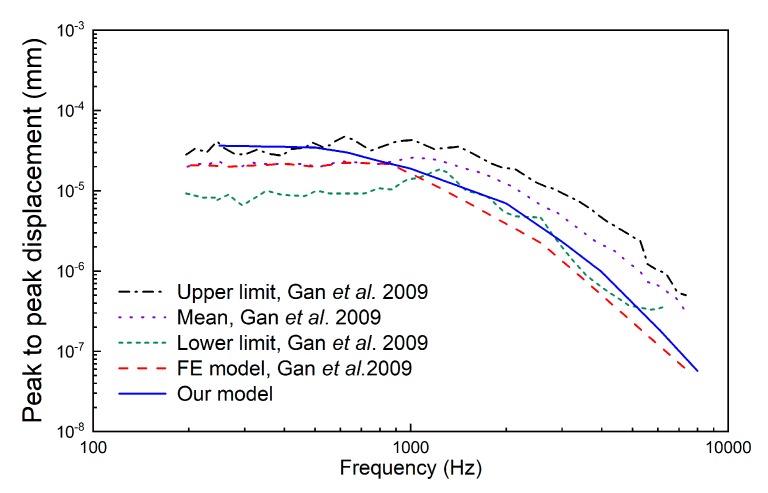
Comparison of stapes footplate displacement under 90 dB sound pressure level (SPL) applied at the eardrum.

**Figure 9:**
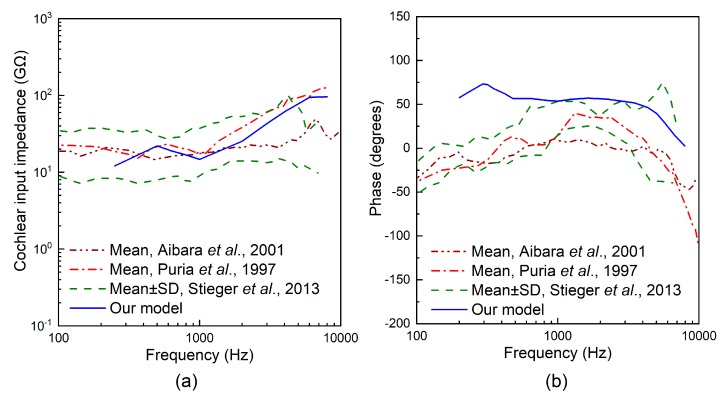
Comparison of the cochlear input impedance: (**a**) magnitude; (**b**) phase.

**Figure 10:**
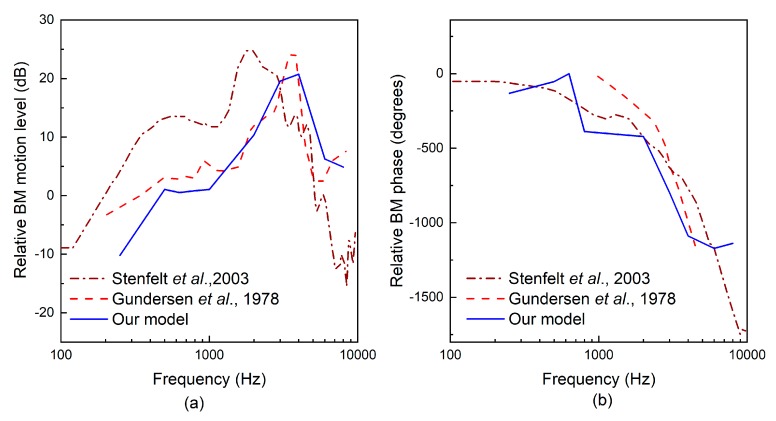
Comparison of the relative basilar membrane motion level at 12 mm from the stapes: (**a**) magnitude; (**b**) phase.

Experimental and theoretical studies have shown that the geometry of the transducer’s tip, which connects the round window membrane and transmits vibration to the cochlea, influences the transducer’s hearing compensation performance [13,23]. To facilitate the design of our coupling rod’s tip, we utilized our previously established human ear finite element model (as shown in Figure 7), which is composed of the middle ear and cochlea. In this paper, we modified the Young’s modulus of some tissues according to literature reports to improve the reliability of this model. The changed parameters and their referenced sources are listed in Table 2. To confirm the validity of the model, we carried out three sets of comparisons with reported experimental results. Firstly, considering that the stapes is responsible for transmitting sound into the cochlea, we calculated its footplate displacement in our model and compared it with Gan et al.’s data [24], as shown in Figure 8. Then, the cochlear input impedance, which also reflects the sound transfer property from the middle ear into the cochlea, was also selected to verify our model. The model-predicted result was plotted with published experimental reports [25,26,27], as shown in Figure 9. Finally, the excited response at a specific place (12 mm from the stapes) on the basilar membrane was calculated and compared with Stenfelt et al. [28] and Gundersen et al.’s [29] measurement data (Figure 10). These comparisons show that our model-predicted results are generally consistent with the experimental data. Thus, we can use this model to aid in the design of this transducer.

**Figure 11:**
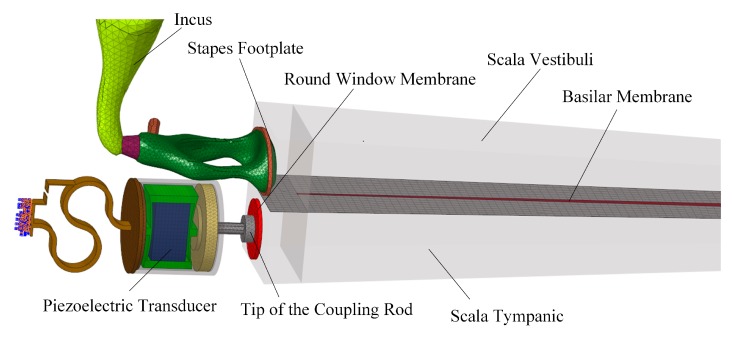
Constructed coupling model of the human ear and the piezoelectric transducer.

**Figure 12:**
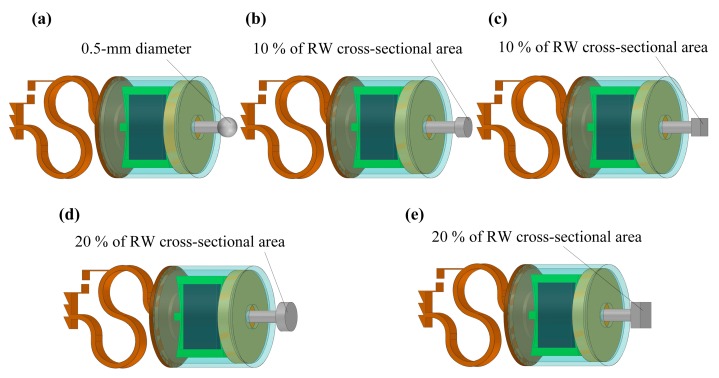
The studied transducer coupling rod’s tips with different geometries: (**a**) 0.5 mm diameter spherical tip; (**b**) 0.5 mm diameter cylindrical tip; (**c**) cubic tip with a 0.44 mm × 0.44 mm square top surface; (**d**) 0.72 mm diameter cylindrical tip; (**e**) cubic tip with a 0.64 mm × 0.64 mm square top surface.

**Figure 13:**
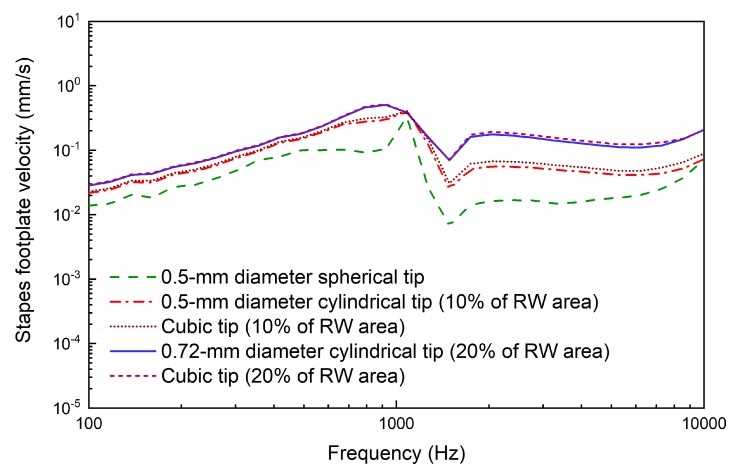
The influence of the coupling rod tip’s geometry on the transducer’s performance (at 0.5 V).

Based on this verified human ear finite element model, we constructed a coupling mechanical model of the human ear and the piezoelectric transducer by coupling the nodes of the transducer’s tip with the attached nodes on the round window membrane. The final constructed coupling model is shown in Figure 11. Then, we changed the coupling rod tip’s geometry and size to study the effect of these parameters on the transducer’s performance. As shown in Figure 12, the following five tips were analyzed: (a) a 0.5 mm diameter spherical tip; (b) a 0.5 mm diameter cylindrical tip; (c) a cubic tip with a 0.44 mm × 0.44 mm square top surface; (d) a 0.72 mm diameter cylindrical tip; and (e) a cubic tip with a 0.64 mm × 0.64 mm square top surface. The influence of these tips on the transducer’s performance is shown in Figure 13. It demonstrates that, with the same diameter, the transducer with the cylindrical tip can stimulate the stapes footplate velocity more than can the transducer with the spherical tip. However, with the same cross-sectional area, the performance of the transducer with the cylindrical tip is similar to that with the cubic tip. Besides this, when the transducer’s tip cross-sectional area increased from 0.2 mm^2^ to 0.4 mm^2^, the transducer-stimulated stapes footplate velocity increased significantly, especially at high frequency. Considering that most sensorineural hearing loss is severe at high frequency and that the cubic tip is more likely to contact the bony surrounding of the round window, we selected the 0.72 mm diameter cylindrical tip, which has a cross-sectional area accounting for 20% of the RW membrane’s cross-sectional area, as our transducer coupling rod’s tip.

### 2.3. Evaluation of the Transducer’s Performance

To evaluate the hearing compensating performance of our designed transducer, we calculated the stapes velocity and compared it with those driven by 94 dB sound pressure level (SPL) sound stimulation applied to the eardrum. Then, the transducer-excited response was converted to the equivalent sound pressure level at the eardrum to generate the same response of the stapes. The corresponding equivalent sound pressure (ESP) of the transducer’s stimulating performance was calculated according to Equation (4):

(4) $$P_{\mathrm{eq}}=94+20\log_{10}(\frac{v_{\mathrm{tr}}}{v_{\mathrm{ac}}})$$

where *v*_ac_ is the sound-stimulated (94 dB SPL) velocity of the stapes and *v*_tr_ is the corresponding transducer-stimulated velocity of the stapes.

## 3. Results

**Figure 14:**
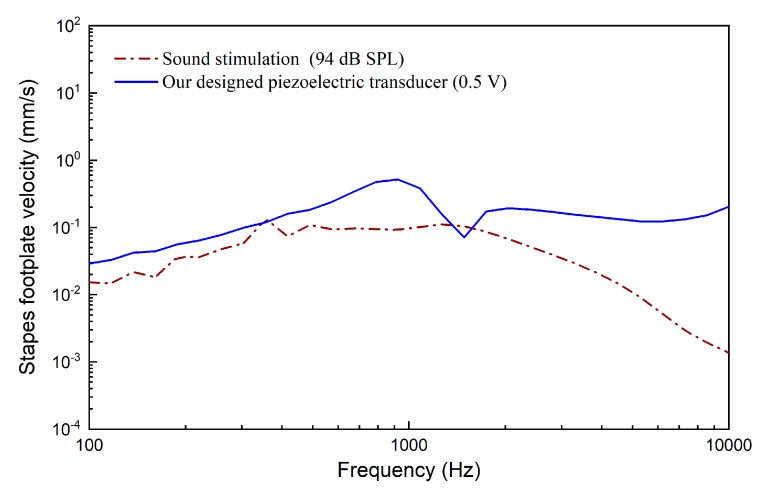
Stapes vibration stimulated by the piezoelectric transducer (0.5 V) and acoustic stimulations (94 dB SPL at the eardrum).

The model-predicted stapes footplate velocity stimulated by the piezoelectric transducer is shown in Figure 14. For comparison, the stapes footplate velocity excited by 94 dB sound pressure applied at the eardrum was also plotted. It demonstrates that the stapes velocity stimulated by this piezoelectric transducer at 0.5 V is similar to that under eardrum acoustical stimulation at 94 dB SPL. Besides this, at high frequency, the piezoelectric-transducer-stimulated stapes vibration is considerably larger than that by acoustical stimulation.

**Figure 15:**
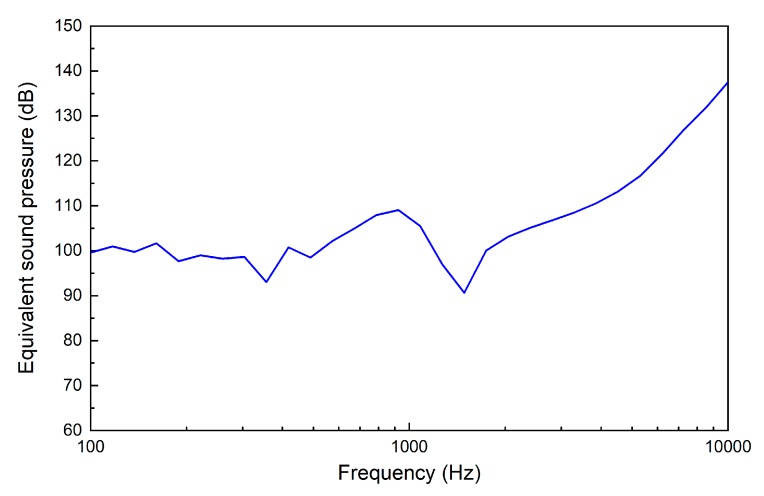
Equivalent sound pressure for the piezoelectric transducer stimulation at 0.5 V.

Figure 15 shows the equivalent sound pressure excited by the piezoelectric transducer at 0.5 V. It indicates that the piezoelectric transducer driven by 0.5 V can generate about 100 dB SPL equivalent sound pressure at the eardrum below 3 kHz, 110 dB at 4 kHz and 120 dB above 6 kHz. Considering many middle ear implant studies use 100 dB SPL as the design criterion, this piezoelectric transducer’s output is adequate. Meanwhile, it also demonstrates that the transducer can compensate severe hearing loss at high frequency, which is a valuable advantage given that most sensorineural hearing loss deteriorates high frequencies more severely than it does low frequencies.

## 4. Discussion

Since human ear is a biology system with a complex structure and ultra-small dimension, systematic experimental study on it for the transducer is difficult to carry out. To aid the design of the transducers in the middle ear implants, many studies utilized human ear finite element models [32,33,34]. Their experimental results have confirmed the validation of this method. Thus, in this paper, we take the same method to optimize our transducer. Furthermore, to ensure the accuracy of our model, we constructed our human ear finite element (FE) model based on high-resolution micro-CT scanning images of fresh human temporal bone. Besides this, we further carried out three sets of comparisons with reported experimental data to verify our FE model’s reliability. Comparison results show that our human ear FE model is reliable and can be used to aid the design of the transducer of middle ear implant.

To treat sensorineural hearing loss in the presence of middle ear disease, in which a transducer cannot couple to the ossicular chain, MED-EL Vibrant Soundbridge’s FMT has been widely implanted on the round window membrane clinically. However, the FMT was not originally designed for RW stimulation. There exists a geometric mismatch between the FMT (with a diameter of 1.8 mm) and the round window membrane (with a diameter of 1.5–1.9 mm [35]). This geometric mismatch would cause the transducer to attach to the bony surrounding of the round window and decrease its transmission efficiency to the cochlea. The tip of our transducer specifically designed for RW stimulation has a diameter of 0.72 mm. This diameter is much less than that of the round window membrane and guarantees the stability of the transducer’s performance. Our study also shows that the piezoelectric transducer’s transmission efficiency is proportional to the cross-sectional area of the transducer’s tip. This result is consistent with that of the experimental report conducted by Schraven et al. [23].

The preload of the transducer on the round window membrane also affects the transducer’s performance and leads to variability in patients’ postoperative outcomes [15,18]. Based on a human temporal bone experiment, Mathias further suggested that the optimal preload of the transducer should be less than 20 mN [16]. However, it is difficult to control the preload during surgery for FMT implantation, since the FMT is fixed just by putting cartilage or connective tissue behind it. Our piezoelectric transducer introduces a force visual indicator with two bars which would coincide when the applied force equals 20 mN. This force visual indicator can help the surgeon to monitor the preload of the transducer during the operation. Besides this, Ishii et al. found that a force of 564 mN applied on the round window membrane would rupture the membrane [36] and lead to further hearing loss. Therefore, the incorporated force visual indicator can also assist the surgeon to prevent the round window membrane’s rupture and protect the patient’s residual hearing.

**Figure 16:**
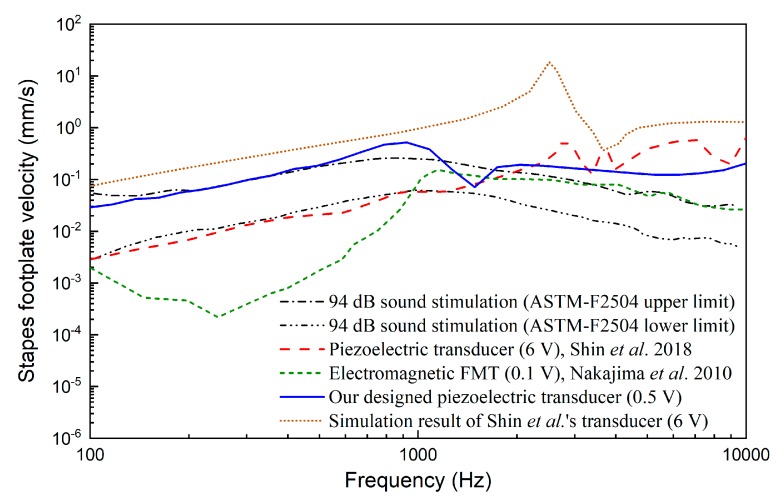
Comparison of stapes responses driven by our piezoelectric transducer and reported transducers.

Hearing loss compensating capability, which should meet the patient’s requirement, is a crucial criterion for middle ear implant design. As previously noted, RW stimulation is a new application of middle ear implants for treating sensorineural hearing loss in the presence of middle ear disease. Since most sensorineural hearing loss is severe at high frequency [2], the transducer should have good performance at high frequency. Besides this, as middle ear diseases (e.g., otosclerosis) are usually worse at low frequency, low-frequency capability is also important for round window stimulation. However, the widely used RW-stimulating transducer, that is, FMT, is designed for stimulating the incus long process and treating sensorineural hearing loss. Clinical reports show that it has a poor output at low frequency [10,37]. In order to study the performance of our designed transducer comparatively, a comparison between our transducer-stimulated stapes velocity with that driven by the FMT is made in Figure 16. In this figure, the FMT-stimulated results were obtained from Nakajima et al.’s experimental data [12], which were measured in the human temporal bone. For comparison, the acoustical-stimulation-excited (94dB SPL) stapes velocities taken from the ASTM-F2504 standard [38] were also plotted in Figure 16. It shows that the FMT-simulated stapes response is similar to that excited by acoustical stimulation at high frequency (>1000 Hz) but significantly smaller at frequencies lower than 1000 Hz, especially at 250 Hz. The poor low-frequency performance would lower patients’ speech perception (vowel sounds) and music perception. In contrast, our designed transducer not only provides a superior output at high frequency but also generates sufficient stapes response at low frequency. This characteristic would benefit the compensation of mixed hearing loss, which has been encountered in the clinical practice of RW stimulation.

**Figure 17:**
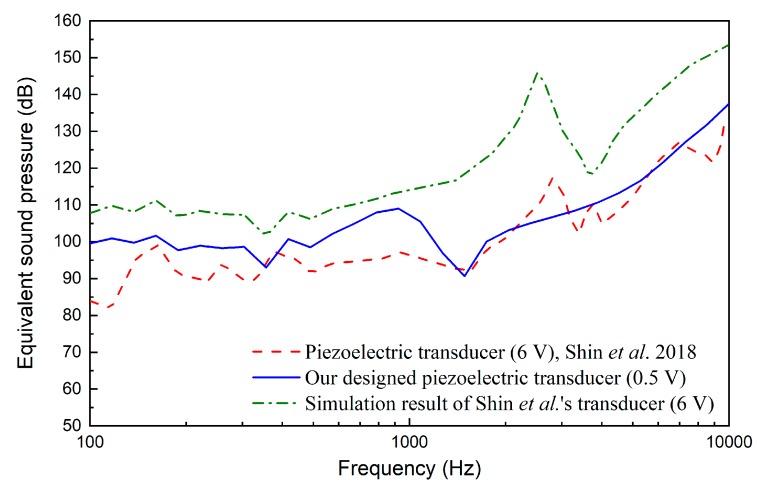
Comparison of the equivalent sound pressures of our piezoelectric transducer and reported transducer.

The performance of the novel piezoelectric transducer specifically designed for round window stimulation by Shin et al. [19] was also selected for comparison. As shown in Figure 16, compared with the FMT, Shin et al.’s piezoelectric transducer also has an excellent low-frequency output. The stapes velocities driven by Shin et al.’s piezoelectric transducer is similar to those excited by normal acoustical stimulation at low frequency (<1000 Hz). Moreover, Shin et al.’s transducer has a better performance at high frequency. Compared with Shin et al.’s results, our designed piezoelectric transducer can stimulate greater stapes response at low frequency. However, it should be noted that the stapes velocities plotted in Figure 16 were derived from different human temporal bones. Thus, comparison of only the transducer-stimulated stapes velocities neglects the individual influence of the temporal bone used. To address this issue, we further calculated the equivalent sound pressure based on the corresponding temporal bone’s acoustical stimulation result, as shown in Figure 17. It demonstrates that under a 0.5 V driving voltage, our piezoelectric transducer can generate an ESP similar to that driven by Shin et al.’s transducer at 6 V.

The above reduction of the required driving voltage is mainly attributed to the improved fixation method of our transducer. Shin et al.’s transducer was fixed by placing dental resin cement on the side of their transducer’s housing; therefore, the coupling condition of the transducer with the round window membrane is difficult to control. Similar to Shin et al.’s transducer, the coupling condition of the FMT is also difficult to control. Investigations of different coupling conditions with FMT in temporal bones, Arnold et al. found that variability between individual results was about 40 dB when using standard-fixation method [39]. To figure out the influence of the transducer’s coupling condition in Shin et al’s experiment, we calculated the ideal stapes footplate velocity excited by Shin et al.’s transducer using our human ear FE model. Since their transducer’s diameter (1.75 mm) is bigger than the diameter of our model’s round window membrane (1.6 mm), we cannot construct a coupling model of their transducer and our human ear FE model. Considering that an ideal piezoelectric transducer is a displacement-driven transducer [40], we stimulated our model’s round window membrane directly using their transducer’s output displacement. The model-predicted results are also plotted in Figure 16 and Figure 17. They show that the simulation result for Shin et al.’s transducer is significantly larger than their experimental result. The fixation method of their transducer introduced a deterioration of 5 dB (at 375 Hz) to 24 dB (at 1395 Hz) in the equivalent sound pressure, which is consistent with Arnold et al.’s results [39].

**Figure 18:**
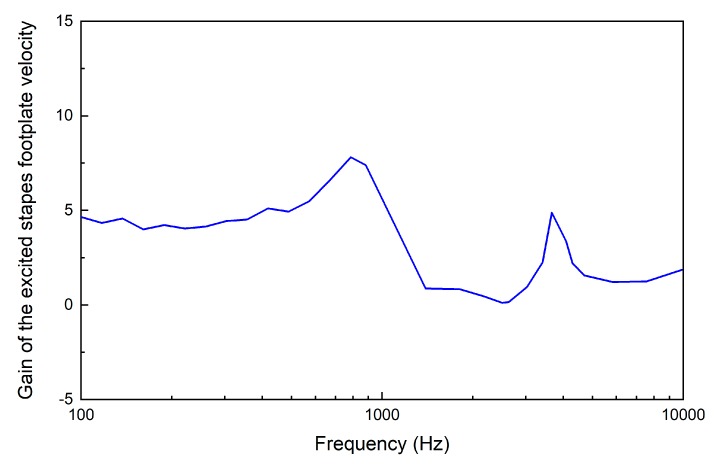
The gain in our transducer-stimulated velocity relative to that stimulated by Shin et al.’s transducer (6 V) [19].

In addition to the improved fixation method of the transducer, the introduction of the flextensional amplifier in our transducer also reduced the required driving voltage and boosted the hearing loss compensating performance. To analyze this improvement, we compared the simulated stapes footplate velocities driven by our transducer and Shin et al.’s transducer under the same driving voltage (6 V). Under this simulation, the coupling condition’s influence of Shin et al.’s transducer was excluded by applied their transducer’s output directly on the round window membrane of our model. The calculated gain in our transducer-stimulated velocity relative to that by Shin et al.’s transducer is shown in Figure 18. It demonstrates that our transducer did amplify the stimulated stapes velocity, especially at low frequency (<1 kHz), with an amplification gain of about 5. This amplification gain value is close to that (5.3) which we aimed for during the design of the flextensional amplifier (in Section 2.2.1).

A middle ear implant is a device implanted in the human ear; therefore, its transducer’s power consumption should be restricted. The piezoelectric transducer’s behavior can be approximated to that of a capacitor when its working frequency is far less than its resonant frequency. As shown in Equation (5), the piezoelectric transducer’s power consumption can be calculated as [5]

(5) $$P_{\mathrm{rms}}=I_{\mathrm{rms}}V_{\mathrm{rms}}\cos\theta =2\pi fCV_{\mathrm{rms}}^{2}\cos\theta$$

where *V*_rms_ is the driving voltage, *f* is the frequency, *C* is the capacitance of the transducer’s piezoelectric stack and cos *θ* is the power factor. Considering that our piezoelectric stack is the same one used by Shin et al., the decrease in the transducer’s power consumption is proportional to the square of the voltage increase. Therefore, the reduction of our designed transducer’s working voltage will significantly decrease its power consumption, which is an advantage for a totally implanted hearing device.

Compared with FMT, our transducer’s structure is relatively complicated. This because of the fact that the FMT was not designed specifically for the RW stimulation; therefore, it does not contain the fixation part fixing the device against the round window and the coupling rod transmitting its vibration to the round window membrane. Nevertheless, compared with other commercial piezoelectric transducers for the middle ear implants, for example, Envoy Medical Corporation’s Esteem [41], our transducer’s mechanical structure is still simple. The key component of our transducer is the piezoelectric component as it responsible for converting the electrical signal to vibration and stimulating the round window membrane. The piezoelectric component consists of two parts: the flextensional amplifier and the piezoelectric stack. The piezoelectric stack, which is used in our transducer, is a commercial product (PAZ-10-0079) produced by Murata Manufacturing Co., Ltd. (Kyoto, Japan). In terms of the flextensional amplifier, its dimensions are compatible to that of the flextensional amplifier fabricated by Wang et al. [42]. Therefore, the mechanical structure of the piezoelectric transducer is acceptable in terms of fabrication.

## 5. Conclusions

Currently, the transducer widely used for round window stimulation (i.e., FMT) has three inherent shortcomings: mismatch between the diameter of the transducer and the RW membrane, uncontrollable preload and poor low-frequency output. To overcome these problems, we proposed a new piezoelectric transducer specifically for round window stimulation. To facilitate the design process of this transducer, a coupling finite element model of the transducer and a human ear was constructed. Then, the transducer’s key design parameters were optimized based on this model. The transducer’s hearing loss compensating performance was also studied and compared with those of the FMT and a recently reported piezoelectric transducer. The results show that our designed transducer generates better output than the FMT, especially at low frequency. Besides this, the power consumption of the transducer was significantly decreased compared with that of a recently reported RW-stimulating piezoelectric transducer.

In subsequent research, we will fabricate the piezoelectric transducer and carry out a human temporal bone experiment to evaluate its feasibility.

## Figures and Tables

**Table 1:** Material parameters of lead zirconate titanate ceramics (PZT-8).

Elastic Stiffness Constant (GN/m^2^)						Piezoelectric Constant (C/m^2^)			Permittivity Constant (× 10^−10^ F/m)	
$c_{11}^{E}$	$c_{12}^{E}$	$c_{13}^{E}$	$c_{33}^{E}$	$c_{44}^{E}$	$c_{66}^{E}$	$e_{15}$	$e_{31}$	$e_{33}$	$\varepsilon_{11}^{s}$	$\varepsilon_{33}^{s}$
146.9	81.1	81.1	131.7	31.4	32.9	10.3	−3.9	14.0	114.2	88.5

**Table 2:** The modified Young’s modulus values in our human ear finite element model.

Components	Young’s Modulus, N/m^2^	Ref.
Incudostapedial joint	4.4 × 10^5^	[30]
Stapedial annular ligament	1.5 × 10^4^	[31]
Lateral mallear ligament	6.7 × 10^4^	[31]
Superior mallear ligament	4.9 × 10^4^	[31]
